# Sporotrichosis: hyperendemic by zoonotic transmission, with atypical presentations, hypersensitivity reactions and greater severity^☆^

**DOI:** 10.1016/j.abd.2021.07.003

**Published:** 2021-12-08

**Authors:** Regina Casz Schechtman, Eduardo Mastrangelo Marinho Falcão, Marciela Carard, Maria Salomé Cajas García, Diana Stohmann Mercado, Roderick James Hay

**Affiliations:** a Instituto de Dermatologia Professor Rubem David Azulay, Pontifícia Universidade Católica do Rio de Janeiro, Rio de Janeiro, RJ, Brazil; b International Foundation of Dermatology, University of London, London, UK

**Keywords:** Mycoses, Neglected diseases, *Sporothrix*, Sporotrichosis

## Abstract

In recent decades, an alarming increase in the number of sporotrichosis cases has been reported in southern and southeastern Brazil, especially in the state of Rio de Janeiro, has been considered a long-term hyperendemic condition associated with feline transmission. According to phenotypic classifications, the *Sporothrix* species recovered from cats were classified as *S. brasiliensis* in 96.5% of the studied cases. This finding has also been demonstrated in humans, which confirms the zoonotic transmission associated with this predominant species in Brazil. The zoonotic transmission of the fungus and its important virulence in the context of the hyperendemic situation in Rio de Janeiro have changed the approach to the disease, which in its classic form was restricted to certain professional groups and very specific regions in the Brazilian territory, into a public health challenge of scientific interest. Its atypical manifestations and hypersensitivity reactions are increasingly frequent, constituting a new sporotrichosis aspect, which deserves attention from the medical community, as well as from other health professionals.

## Introduction to the disease and predominant species in Brazil

Sporotrichosis, described in 1898 by Benjamin Schenck,^1^ is a subcutaneous or implantation mycosis, of subacute or chronic evolution, whose agents are dimorphic fungi of the genus *Sporothrix*.^2^, ^3^

Although more frequent in tropical and subtropical climates, especially in humid environments, the disease has been described around the world, with case series published in all continents and in different climates and populations. Fungi of the *Sporothrix* genus are found in nature, mainly in the soil. The infection usually results from the fungus inoculation through thorns, wood splinters, or small injuries caused by activities such as agriculture, gardening, hunting, horticulture, and carpentry, among others^2^, ^3^ or by zoonotic transmission, with the latter form gaining notoriety in recent decades.^4^ The most relevant pathogenic species are *Sporothrix schenckii sensu stricto*, *S. brasiliensis*, *S. globosa* and *S. mexicana*.^5^

The zoonotic transmission is more frequently related to dogs and felines, with domestic cats being the main reservoirs, showing positive cultures in samples from skin lesions, nasal cavity, oral cavity, and nails, confirming the hypothesis of transmission either through animal scratch or bite, as well as by contact with secretions full of infecting microorganisms.^3^

In recent decades, an alarming increase in the number of cases has been reported in the southern and southeastern regions of Brazil, mainly in the state of Rio de Janeiro, and is considered a long-term hyperendemic condition associated with transmission by felines.^4^ The *Sporothrix* species recovered from cats were identified as *S. brasiliensis* in 96.5% of the studied cases. This finding has also been demonstrated in humans, which confirms the zoonotic transmission associated with this predominant species in Brazil.^4^, ^6^

In the murine model study conducted by Arrillaga-Moncrieff et al. in 2009, *S. brasiliensis* was described as the most virulent species in terms of mortality, tissue damage and systemic spread among the studied species (*S. globosa* and *S. mexicana*).^7^ *S. brasiliensis* has been linked with atypical presentations such as disseminated sporotrichosis in immunocompetent patients, conjunctival or mucosal involvement, and hypersensitivity reactions.^6^ Despite its greater virulence, *S. brasiliensis* does not seem to have greater resistance to antifungals, as its growth can be inhibited by low concentrations of itraconazole,^8^ the drug of choice, with shorter duration treatments.^7^ The zoonotic transmission of the fungus and its significant virulence in the context of the hyperendemic situation in Rio de Janeiro have changed the approach to the disease, which in its classic form was restricted to certain professional groups and very specific regions in the Brazilian territory, into a public health and scientific interest public health challenge of scientific interest, since atypical manifestations and hypersensitivity reactions are increasingly frequent, constituting a new sporotrichosis aspect, which deserves attention from the medical community, as well as from other health professionals.

## Epidemiology - peculiar aspects of the zoonotic transmission

Sporotrichosis is a universal mycosis which occurs all over the world, but it is endemic mainly in regions with a tropical and subtropical climate. Occasionally, outbreaks or epidemics occur, usually of short duration. It is classified as an implantation mycosis, as its transmission route is classically described as the inoculation of the fungus into the subcutaneous tissues by trauma in individuals who work with plant and soil handling plants and soil.^3^

The zoonotic transmission has been described as involving cats, dogs, rats, squirrels, armadillos, and birds.^3^, ^4^, ^8^, ^9^ The *S. brasiliensis* species is associated with zoonotic transmission through minor trauma, usually through the cats scratches and bites. Cats remain infected, some asymptomatic, for many months, serving as a reservoir for the fungus. Cat-cat and cat-human transmission perpetuates the spread, causing the infection to expand territorially.^4^, ^8^

Inter-human transmission is rare and unlikely due to the small amount of fungus present in the lesions.^3^ However, repeated and close exposure to the open lesion has been reported as a possible form of inter-human contagion.^10^, ^11^

Sporotrichosis in cats was first described in Brazil in the 1950s.^12^, ^13^ In 1955, Almeida et al. described a possible case of a cat scratch as being responsible for fungus inoculation in a patient in São Paulo.^14^ In 1989, Larsson et al. described a case, from the north coast of São Paulo, of sporotrichosis in a feline being transmitted to three people through scratching.^15^

Currently, there are case reports in felines and humans in almost every state in Brazil^4^, ^16^, ^17^, ^18^, ^19^, ^20^, ^21^, ^22^, and cases of zoonotic transmission have already been reported in other Latin American countries, such as Argentina, Paraguay, and Panama.^23^ Less urbanized states, where the classic form of transmission by *S. schenckii* has been described for years, with great relevance in these areas, such as Amazonas and Pará, also seem to be experiencing a change in clinical and epidemiological presentations.^22^, ^23^ Sporotrichosis, in recent years, has been responsible for hospital admissions and deaths throughout Brazil.^24^

Rio de Janeiro is the state with the highest number of cases reported in the literature, and zoonotic transmission by felines has been described in this state since the 1990s,^4^ with a progressively more severe nature in recent decades especially when there is coinfection with HIV/AIDS.^25^, ^26^

In 2010, Silva analyzed the spatial distribution of human sporotrichosis in the state of Rio de Janeiro, based on data from 1,848 patients treated at the National Institute of Infectious Diseases (INI - *Instituto Nacional de Infectologia*/Fiocruz), between 1997 and 2007, and described a concentration of cases, thus characterizing a possible “sporotrichosis belt” on the borders between the capital city and municipalities in the lowlands of Rio de Janeiro, a less urbanized region.^27^ Later, a new analysis at the same research center suggested the progressive spread of this mycosis to the remainder of the municipality, particularly to the western area and also to all regions of the state of Rio de Janeiro.^28^

Until 2011, epidemiological data in this state came from only a few publications, mainly from the INI/Fiocruz group. In 2011, the notification of the disease was started, and in 2013 sporotrichosis was classified as a notifiable disease throughout the state of Rio de Janeiro, and care was decentralized.^29^ This measure was of paramount importance for epidemiological surveillance improvement and the implementation of measures to control sporotrichosis, in addition to providing subsidies for conducting clinical and epidemiological studies that allow the understanding of the spatial dynamics of the disease over the years.^28^

**Figure 1:**
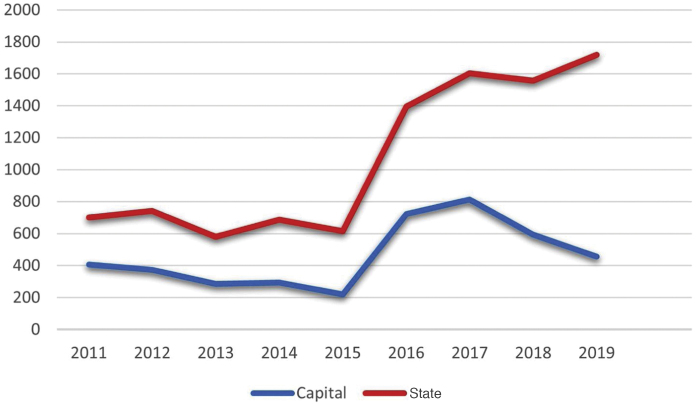
Number of sporotrichosis cases reported in the state of Rio de Janeiro and in the capital city from 2011 to 2019. (Chart prepared by the authors. Source: SES-RJ 2020).

Considering the notifications made to the State Health Secretariat of Rio de Janeiro, the number of cases from January 2011 to September 2020 totaled 10,313 (Fig. 1). The capital concentrated 42.4% (4,369) of the notifications in the same period. The total number tends to be underestimated, considering the underreporting, underdiagnosis, and some cases of spontaneous cure before medical care is attained.

In 2019, 1,720 cases were reported throughout the state of Rio de Janeiro, which corresponds to an incidence of 9.96 cases per 100,000 inhabitants. It is possible to observe a dissociation in the increase in the notifications from the state and the capital city from 2015 onwards, possibly representing the internalization of the hyperendemic situation, beyond the previously described limits. The decrease in notifications in the capital from 2017 onwards should be analyzed in more detail in future studies, as it does not seem to be in accordance with what is reported in the literature and the authors’ clinical practice.

Other Brazilian states (Pernambuco, Minas Gerais, Paraíba) and municipalities also started compulsory notification after the emergence of outbreaks of cases in felines and humans.^24^ In February 2020, an ordinance of the Ministry of Health determined the inclusion of the disease in the national list of compulsory notification of diseases, injuries and public health events.^30^ However, in the same year, the measure was revoked.^31^

To control this hyperendemic situation, more robust measures are required from the government with the collaboration of the health sector and the general population, taking into account the “One Health” approach. Spaying/neutering and early treatment of cats could reduce transmission between animals and from animals to humans. Additionally, educational measures to ensure responsible ownership and use of personal protective equipment could contribute to the prevention of zoonotic transmission. The investment in studies for the development of a sporotrichosis vaccine for animals and/or humans should also be encouraged as a control measure for hyperendemics.^23^ Moreover, the inclusion of mycosis in the lists of neglected diseases would provide more evidence for this hyperendemic undergoing constant expansion, generating greater resources for large scientific studies aiming to contain the spread of the disease.

In the current situation of a Sars-CoV-2 pandemic simultaneously with a sporotrichosis hyperendemic, the authors observed delay in the diagnosis of sporotrichosis, having treated several patients with months of disease evolution. Delay in treating the disease triggers an increase in the number of secondary infections and can even lead to more severe sequelae due to the longer duration of the disease. The authors emphasize the importance of teledermatology at the beginning of treatment, in the management of these patients, and in advising colleagues in primary care. Studies are needed to assess the real impact of the pandemic on sporotrichosis.

## Clinical aspects - classic forms, coinfection with HIV, atypical forms, and hypersensitivity phenomena

Sporotrichosis is a multifaceted disease that can mimic other dermatoses. Variations in its clinical spectrum have been attributed to factors such as mode of inoculation, inoculum size and depth, host immunity, virulence, and heat tolerance of the strain/lineage.^3^, ^6^, ^32^ Sampaio and Lacaz proposed the classification of the disease into four categories: cutaneous lymphatic (the most common form), followed by localized cutaneous (20%), disseminated (cutaneous or systemic), and extracutaneous (mucosal, bone, ocular, articular, visceral) types.^33^ There are other equally valid classifications with minor differences, but the authors chose to adopt that classification.

**Figure 2:**
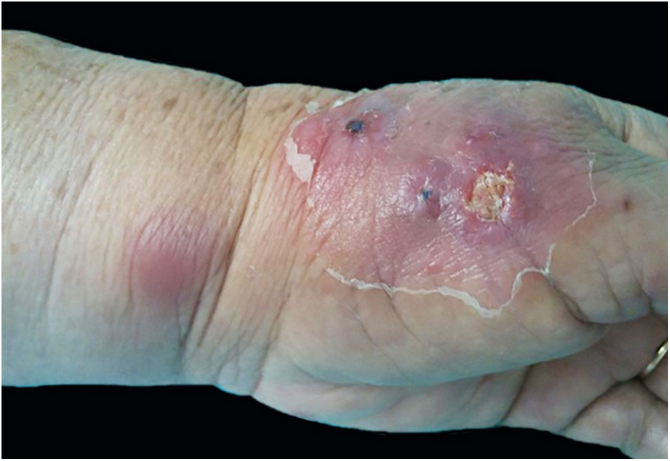
Cutaneous-lymphatic sporotrichosis. Sporotrichosis inoculation ulcer on the back of the right hand.

**Figure 3:**
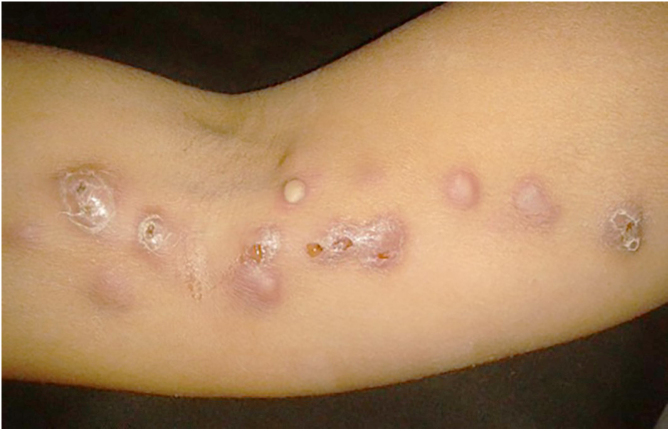
Cutaneous lymphatic sporotrichosis. Nodules draining seropurulent secretion, papules and pustules on the right upper limb along the ipsilateral lymphatic chain.

**Figure 4:**
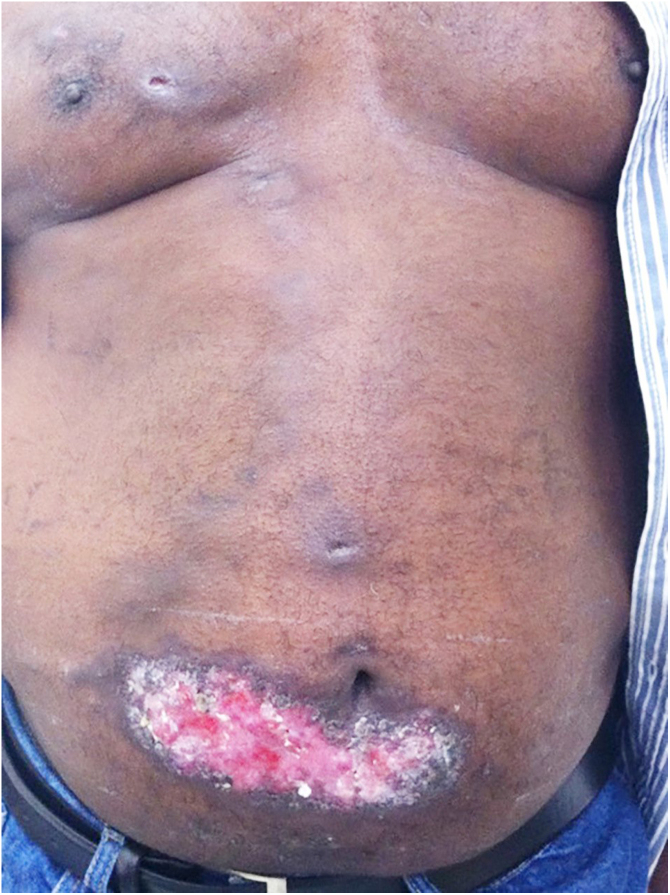
Cutaneous lymphatic sporotrichosis – aggressive presentation. Extensive ulceration in the lower abdomen and nodules along the lymphatic chain of the anterior trunk.

**Figure 5:**
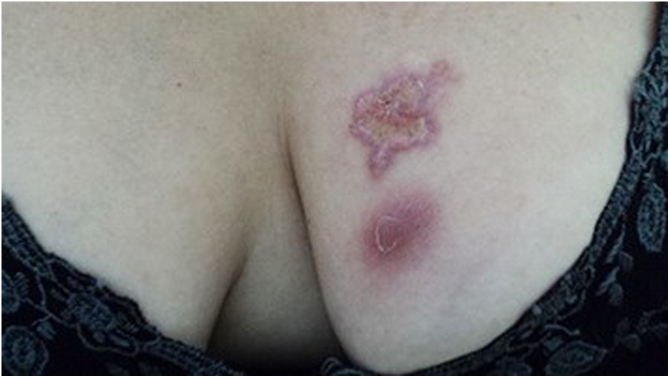
Cutaneous lymphatic sporotrichosis. Ulcerated plaque on the left breast with erythematous borders and an infiltrating nodule just below the plaque.

The cutaneous lymphatic type (Figure 2, Figure 3, Figure 4, Figure 5) is the most frequent one, occurring in more than 75% of cases. It mainly affects the hands and forearms in adults, while in children, the face is a common site, accounting for more than 92% of pediatric cases. It is initially characterized by a papule, nodule or gumma that ulcerates (inoculation ulcer), and then new nodules or gumma appear along one or even more than one lymphatic path to the regional lymphatic chain (centripetal lymphatic route). Mild regional adenopathy may occur, and systemic symptoms, when present, are generally mild. Bilateral disease is rare, and when it occurs, it suggests multiple foci of inoculation. In the hyperendemics due to zoonotic transmission in Rio de Janeiro, the inoculation of more than one site by cat scratches or bites is common.^4^

**Figure 6:**
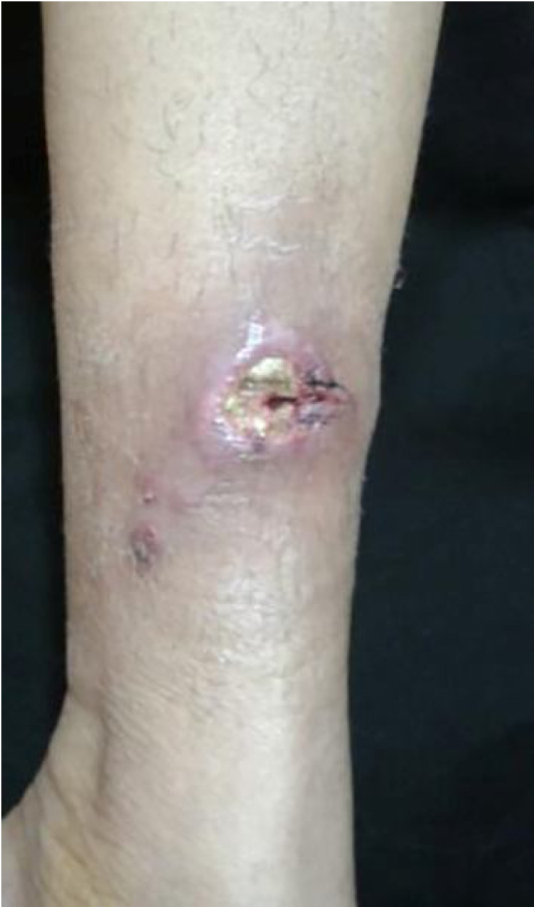
Fixed cutaneous sporotrichosis – ulcerated solitary plaque, with erythematous borders and a serous base.

The localized or fixed cutaneous type mainly affects the face, neck, trunk, and legs, with the face being the preferred site (Fig. 6).^33^ Generally, there is no lymphatic dissemination during the course of the disease, which shows the host’s good immune response, and the lesion is restricted to the inoculation site. It is characterized by a papular, papulotuberous lesion or verrucous plaque, with or without ulceration; it may or may not be accompanied by satellite lesions. These presentations with “satellite” or “herpes-like” lesions tend to be chronic and usually do not cure spontaneously. Lesions may also resemble keratoacanthoma, facial cellulitis, pyoderma gangrenosum, nodular prurigo, lupus vulgaris, soft tissue sarcoma, basal cell carcinoma, carcinoma erysipeloide, or rosacea. Moreover, the verrucous presentation requires the differential diagnosis with paracoccidioidomycosis, leishmaniasis, chromomycosis, and tuberculosis (known by the acronym “PLECT”).^33^, ^34^

The disseminated type is rare and can be either cutaneous or systemic. Both forms result from the inoculation, ingestion or inhalation of the fungus. The first (disseminated cutaneous sporotrichosis or DCS) is characterized by multiple papule-pustular-follicular-crusted lesions associated with plaques or ulcers in non-contiguous sites, in any area of the cutaneous tegument, without extracutaneous involvement. It occurs through hematogenous dissemination.

Systemic disseminated sporotrichosis (SDS) is characterized by two or more affected systems - mainly the central nervous system (CNS), lungs, osteoarticular system, and bone marrow – in addition to fever and impairment of the overall status. It is worth recalling that there are disputes in the scientific community regarding the division of the disseminated type into cutaneous (DCS) and systemic (SDS) forms since internal organ involvement can occur in most cases.^34^, ^35^, ^36^

It is common knowledge that the disseminated presentation is almost always associated with some type of immunosuppression (alcoholism, diabetes mellitus, sarcoidosis, tuberculosis, transplant recipients, malignancy, immunosuppressive therapies, AIDS).^34^ In a retrospective study (1990–2015) that included 24 cases of DCS, Bonifaz et al found decompensated diabetes and chronic alcoholism as the most frequent predisposing factors for this clinical form of sporotrichosis. These conditions justify the immune deterioration related to the cellular immune response, especially macrophage dysfunction.^37^

The extracutaneous type is rare, difficult to diagnose due to the absence of skin lesions, and is often associated with spore inhalation and hematogenous dissemination in immunosuppressed patients. Bones are the structures most frequently affected, accounting for 80% of cases of extracutaneous disease, especially the tibia, hand bones, radius, and ulna. Articular involvement manifests mainly through monoarthritis with edema, synovial effusion, and functional limitation, which can affect knees, elbows, ankles or wrists, the first being the most commonly affected site. It is important to insist on early diagnostic proof by synovial fluid culture or synovial biopsy, as osteomyelitis can occur if there is no adequate treatment of the infection.^34^, ^35^

Pulmonary sporotrichosis is rare and clinically similar to tuberculosis. It can present as a primary infection through the inhalation of spores, and a cavitary or disseminated/non-cavitary pattern. The primary pulmonary form is the most common due to the inhalation of conidia. Hilar lymphadenopathy and pleural effusion may be present. Cytological analysis and culture of the induced sputum reveal the pathogen in most cases.^38^

Sporotrichosis with CNS involvement is extremely rare, associated in most cases with immunosuppression. Cerebrospinal fluid (CSF) examination shows pleocytosis, hyperproteinorrhachia, and hypoglycorrhachia. CSF culture is mandatory to establish the correct diagnosis.^39^

The mucosal location can be primary due to direct inoculation (primary mucosal sporotrichosis or PMS) or secondary to the disseminated form, with PMS being the most common form. Although any mucous membrane can be affected by sporotrichosis, the ocular mucosa is most commonly affected, causing episcleritis, uveitis, choroiditis, retrobulbar lesions, and conjunctivitis, which, when accompanied by regional lymphadenopathy, is called Parinaud's oculoglandular syndrome, which is not uncommon. When the tear duct is affected, dacryocystitis can occur as a consequence.^35^

### Sporotrichosis and HIV coinfection

Although HIV-infected patients are at increased risk of developing other deep and disseminated fungal infections, sporotrichosis is relatively less frequent in these patients than other mycoses. When coinfection occurs, it is more often disseminated and the patient has a very low CD4+ T-cell count.^36^ The clinical picture of sporotrichosis in the AIDS patient is quite variable, resulting in ulcers, acneiform lesions, indurated plaques, or crusts.^34^, ^39^, ^40^ In addition, systemic manifestations may develop, which include severe bone lesions, lung and spleen involvement, in addition to meningitis, mainly due to neurotropism related to *S. brasiliensis*, leading to sepsis and death. Interestingly, despite being a vulnerable group, reports of SDS in transplant patients are not as frequent.^41^

At a time when the use of immunobiologicals for several autoimmune diseases is on the rise, it is important to consider the occurrence of a hyperendemic and advise professionals and patients regarding contact with sick animals and, when possible, wait for the end of the mycosis treatment before starting medications that can cause immunosuppression.

It is important to highlight that sporotrichosis can present as an immune reconstitution inflammatory syndrome (IRIS) during antiretroviral therapy for the treatment of AIDS or be the first manifestation of immunosuppression in the disseminated form.^25^, ^39^

An increase in the number of cases, hospitalizations, and deaths of HIV-coinfected patients with sporotrichosis has been documented in Rio de Janeiro, which can be explained by an acceleration in the spread of sporotrichosis in urban areas in recent years, affecting more of this vulnerable population.^25^, ^26^ This emphasizes the importance of broader HIV screening and of professional increasingly alert of the possibility of coinfection and the severity of this condition.

Freitas et al retrieved information from 3,618 patients diagnosed with sporotrichosis, 48 ​​of which co-infected with HIV, registered between 1987 and March 2013 at INI/Fiocruz, a national referral center for infectious diseases. This study showed that HIV infection with a low CD4+ cell count aggravates sporotrichosis, since these patients had a more severe disseminated disease, 42-fold greater need for hospitalization, and 45-fold greater risk of death from this mycosis than individuals not infected with HIV. Due to its aggressive presentation, sporotrichosis led to HIV testing and subsequent diagnosis in 19/48 patients.^25^ This is due to the fact that HIV clearly modifies the natural history of sporotrichosis and is associated with a broad spectrum of presentations of this mycosis. CD4+ T cells play a crucial role in the control of sporotrichosis, and these cells are exactly the main target of HIV infection.

In another retrospective search in the INI/Fiocruz database between 1999 and 2015, 3,917 patients were diagnosed with sporotrichosis, 75 of whom were hospitalized and 11 died. HIV infection was present in 29 patients (38.7%), with a mean CD4+ cell count of 52 cells/μL. The hospital length of stay was longer in patients with sporotrichosis/HIV infection, as well as the Odds Ratio for hospitalizations (55.7; 95% Confidence Interval [95% CI]: 32.0–96.9) and deaths (69.8; 95% CI: 20.8–234.5) when compared with non-HIV-infected individuals.^26^

### Atypical forms

In recent years, after several changes in the taxonomy of *Sporothrix schenckii* and with the advent of the zoonotic hyperendemic situation in Rio de Janeiro, new observations on the clinical aspects of sporotrichosis have emerged, which the authors of this article call the “new facet of sporotrichosis”. Several presentations, hitherto uncommon, were identified. In this context, different species of the *Sporothrix* complex seem to be associated with certain clinical manifestations of sporotrichosis and atypical presentations of the mycosis.^6^, ^7^

In the zoonotic hyperendemic in Rio de Janeiro that has occurred since the 1990s, in which contact with infected felines is the main source of transmission, patients who are infected with *S. brasiliensis* have a higher number of statistically significant hypersensitivity reactions and disease dissemination.^4^, ^42^, ^43^, ^44^, ^45^, ^46^, ^47^

In a cross-sectional study of 50 patients with different clinical forms of sporotrichosis, Almeida-Paes et al. revealed a distinct clinical picture in infections caused by *Sporothrix brasiliensis* when compared to patients with *S. schenckii*. *S. brasiliensis* was associated with the disseminated cutaneous infection without underlying disease, hypersensitivity reactions, and mucosal infection, while patients with *S. schenckii* had less severe and more frequently localized disease, demonstrating a direct association between unusual clinical presentations of human sporotrichosis, including severe disease in immunocompetent individuals, with infection by *S. brasiliensis*,^6^ suggesting a greater virulence of the latter.

### Sweet’s syndrome

Sweet’s syndrome presents with erythematous plaques or nodules and, on histopathology with a dense neutrophilic infiltrate without leukocytoclastic vasculitis. It may be accompanied or not by fever and leukocytosis with neutrophilia, and a good response to corticosteroids. Recently, de Lima et al. described 10 cases of Sweet’s Syndrome in patients with sporotrichosis. These patients met the histopathological criteria for the diagnosis, in addition to having a culture compatible with *Sporothrix sp*. After careful anamnesis, the possibility of a drug reaction was ruled out. The incidence of this syndrome in the 379 patients with sporotrichosis was 2.9%. All studied cases of Sweet’s syndrome were associated with infection by *S. brasiliensis*.^43^ In another case series published in 2011, Sweet’s syndrome was more frequent in adult female patients, who were successfully treated with steroids and itraconazole.^44^

### Erythema nodosum

**Figure 7:**
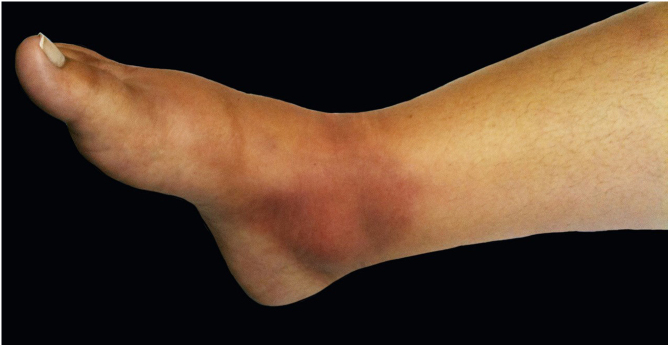
Hypersensitivity phenomenon. Erythema and edema over the medial malleolus compatible with erythema nodosum.

Erythema nodosum is characterized by the sudden appearance of erythematous nodules in the anterior region of the lower limbs, accompanied by fever, arthralgia, and myalgia. This clinical syndrome may be secondary to sporotrichosis (Fig. 7). This is probably due to a mechanism of hypersensitivity to infection as described in other mycoses, mainly systemic, such as coccidioidomycosis, histoplasmosis, and paracoccidioidomycosis. In these cases, the presence of erythema nodosum represents a good prognostic factor. In 2002, Gutierrez-Galhardo et al. presented a series of three cases of erythema nodosum associated with sporotrichosis and zoonotic transmission in the state of Rio de Janeiro. Two of the patients had nodules accompanied by ulcerated lesions in the lower limbs, and one of them had only nodules. In all of them histopathology was compatible with erythema nodosum.^42^ Another case published in 2013 described a 28-year-old, immunocompetent female patient who had erythematous nodules in her lower limbs, associated with typical sporotrichosis lesions confirmed by culture. The condition was resolved with the use of non-steroidal anti-inflammatory drugs and itraconazole.^45^

### Erythema multiforme

Erythema multiforme is an acute disease, usually localized, presenting with lesions (single or multiple) that affect the skin in its minor form and the mucosa in its major form. It is associated with an inflammatory response related to infections in 90% of cases, with herpes simplex virus type 1 being the most frequent etiology, followed by herpes simplex type 2 and *Mycoplasma pneumoniae*. In 10% of cases, it is associated with the use of medications, especially non-steroidal anti-inflammatory drugs.^45^

**Figure 8:**
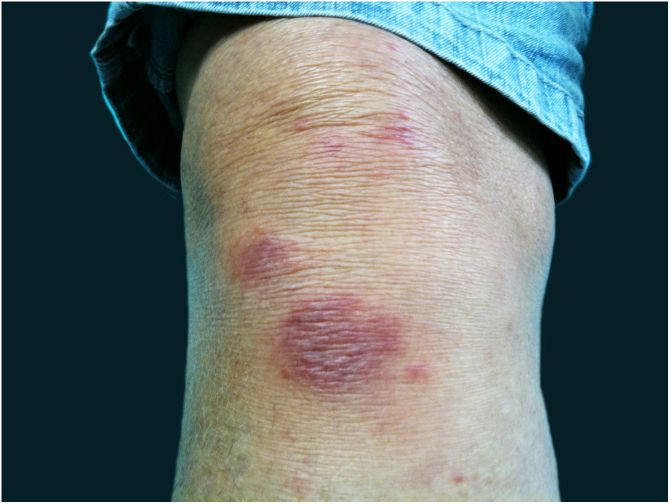
Hypersensitivity phenomena. Fixed cutaneous sporotrichosis with erythema multiforme hypersensitivity phenomenon.

Erythema multiforme has been associated with infections by histoplasmosis, coccidiomycosis, and some cases of dermatophytosis and sporotrichosis (Fig. 8). The diagnosis is based on the presence of clinically typical erythema multiforme and histopathological identification of fungi.^46^

### Arthritis

Osteoarticular involvement is the most common form of extracutaneous sporotrichosis, commonly through traumatic inoculation of contaminated materials into the skin. The bone-related forms are generally associated to immunosuppressed patients, such as alcoholics or those with HIV/AIDS.^48^ Arthritis can present as a hypersensitivity reaction associated with sporotrichosis in individuals without immunosuppression. Orofino et al. reported the case of a previously healthy patient who, after contact with a sick cat, had edema, erythema and pain on mobilization in both elbows, associated with ulcerated lesions and a positive culture for *Sporothrix*. The patient had routine laboratory tests within normal parameters, with the exception of the ESR, which was slightly elevated, whereas the radiography showed no alterations. After treatment with potassium iodide, the lesions disappeared, as did the articular involvement.^49^

Arthritis in sporotrichosis is usually monoarticular, often affects the knee, wrist, elbow, or ankle, and presents with edema and inflammatory signs, usually without systemic symptoms. Laboratory tests are usually normal, with the exception of ESR. Ultrasonography and radiography also show no alterations. The diagnosis is based on the anamnesis, clinical manifestations, and response to antifungal treatment, taking into account that the gold standard for diagnosing sporotrichosis is the fungus isolation from material extracted from a lesion.^48^

Hypersensitivity reactions in sporotrichosis are probably related to frequent contact with fungal antigens in the environment, related to the hyperendemics in the state of Rio de Janeiro.^6^ This constant exposure could lead to intense stimulation of the immune system in individuals who are frequently in contact with infected cats and who tend to have a large number of fungal cells in the lesions.^5^ Each exposure can generate a trigger and culminate in subclinical infection or reinfection and cause hypersensitivity. It is not yet known whether the hypersensitivity reaction is conditioned by the response of the host to infection, genetic predisposition, inoculum size, or strain virulence.^6^, ^7^

It was also observed that patients who had hypersensitivity reactions had a faster resolution of sporotrichosis than the patients who did not. Of these, a large percentage had the fixed cutaneous form. This may mean that hypersensitivity reactions denote an increased immune response of these patients to *S. brasiliensis* (since the fixed form is due to good resistance) and possibly a protective role, as seen in coccidioidomycosis.^6^, ^7^

Therefore, in-depth studies are needed, associating molecular biology and including the clinical aspects of the infection caused by *S. brasiliensis*, aiming to develop new species-specific therapies and, not less important, directed to the immunological aspects of circulating species, contributing to disease prophylaxis through vaccination of animals and humans.

## Diagnosis – mycological examination, histopathology, serological tests and molecular tests

In a hyperendemic situation, with few available resources, the clinical and epidemiological diagnosis is acceptable to initiate treatment. However, the definitive diagnosis depends on the confirmation of the fungus present in the sample.

Classic, low-cost, and accessible exams include direct microscopic examination (DME) and culture of the material collected such as samples and liquid exudates from skin lesions. In cases of extracutaneous involvement, specimens such as sputum, urine, blood, synovial fluid, and cerebrospinal fluid may be analyzed.^50^

DME with 10% potassium hydroxide or with dimethyl sulfoxide allows observing the “cigar-shaped” yeast cells with an optical microscope; however, these structures are difficult to be observed due to the paucicellularity and small diameter (2– 6 µm) of the fungus. This test has a low specificity because there are other morphologically similar yeast fungi.^50^

The culture is more sensitive and specific than DME. It should be performed on Sabouraud glucose or Mycosel agar at 25 °C. The time of growth is variable and the fungus becomes visible after three to seven days, but some samples take longer to grow, between 4 and 5 weeks.^3^, ^50^ In daily practice, the authors recommend not discarding the culture tube within a period of less than 30 days after observing that certain samples from Rio de Janeiro took longer to grow.

**Figure 9:**
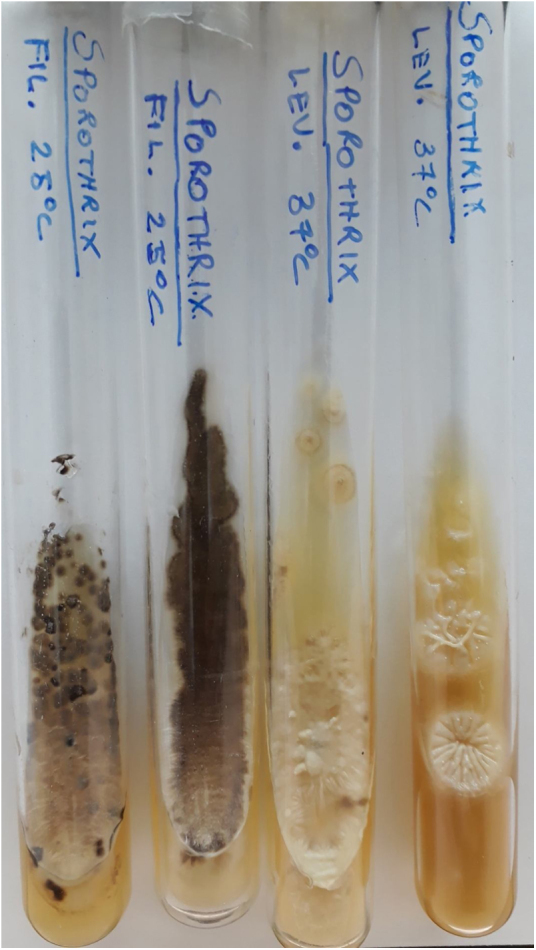
Culture tubes with Agar Sabouraud. Filamentous form (25 °C) with gray to black membranous colonies. Yeast form (37 °C) with beige creamy colonies forming ridges from the center outward.

The initial colonies are smooth and humid, cream-colored with a darker center. Over time, usually around seven days, they darken, and become brown to black due to the production of melanin characteristic of dematiaceous fungi (Fig. 9). It is important to emphasize that this fungus is not considered a dematiaceous fungus, although it may produce black pigment with maturity.^3^

**Figure 10:**
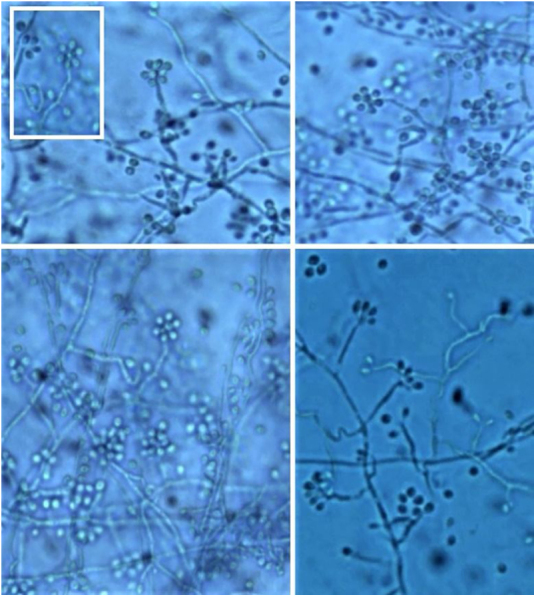
Microscopy of culture (filamentous form 25 °C) – Thin, hyaline hyphae with conidiophores implanted at 90° from the hyphae and terminal phialides in a characteristic floral arrangement or “daisy-like” shape.

It is necessary to carry out the micromorphological study to recognize the filamentous forms that grew at room temperature (25 °C), observing septate and branched mycelium with thin, hyaline hyphae, reproducing through unicellular, oval or piriform conidia arranged along the hyphae shaped like bunches or a daisy-like floral arrangement (Fig. 10).^3^

However, cultures at 35–37 °C need enriched media, such as brain and heart infusion agar, blood or chocolate agar for colonies to grow for 5 to 7 days. They have a creamy yellow or brown aspect. On the microscopy of the culture, one can identify yeasts reproducing by budding. This conversion of the conidia-producing fungus of the filamentous form at 25 °C (at room temperature) to yeast at 37 °C (in host tissues) confirms the diagnosis.^3^

Although the culture is considered the gold standard test for the diagnosis of sporotrichosis, it does not have the capacity to differentiate between species of the *Sporothrix* complex, which have different virulence and susceptibility to antifungal treatments. Arrillaga-Moncrieff et al. observed that *S. brasiliensis* and *S. schenckii* are the most virulent species compared to *S. globosa* and *S. mexicana*, which demonstrated little or no virulence in the murine model.^7^ However, in more severe cases or those with poor therapeutic responses, more specific tests would be required to differentiate between diverse species.^51^, ^52^

### Histopathology

The anatomopathological study of tissues infected with sporotrichosis has the same limitations as DME, as the few fungal elements presents are small, making their detection difficult, with low sensitivity. In order to increase the detection of fungal structures, periodic acid of Schiff (PAS) or Grocott’s methenamine silver staining are used, which facilitate the observation of yeast cells shaped like a “cigar”. Furthermore, the absence of fungi is related to the host’s response and the time of evolution. In more acute cases, histopathology demonstrates extensive inflammation with neutrophils or abscesses and a predominance of macrophages; however, more chronic lesions or with a better-developed immune response are characterized by the formation of epithelioid or tuberculoid granulomas, presence of lymphocytes, caseous or fibrinoid necrosis, and fibrosis.^51^, ^53^

**Figure 11:**
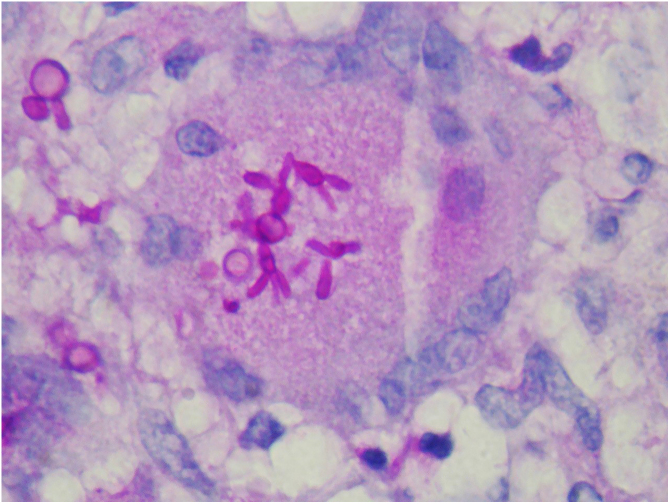
Histopathological examination of the skin of a patient with the disseminated form. “Cigar-shaped” yeast cells (PAS, ×400).

The finding of asteroid bodies or Splendore-Hoeppli phenomenon consists of an eosinophilic material surrounding the fungal cell, probably representing immunoglobulins adherent to the microorganism wall; however, it is not specific for sporotrichosis (Fig. 11).^53^

### Serological tests

Serological tests facilitate the diagnosis and follow-up of cases with systemic manifestations where the collection of material for analysis is difficult or in atypical forms of sporotrichosis when the diagnostic suspicion is strong, and the culture is constantly negative. The first techniques of immunoelectrophoresis, agglutination and immunodiffusion using antigenic fractions were poorly sensitive and specific and have been replaced by immunoenzymatic tests, especially ELISA (Enzyme Linked Immuno Sorbent Assay) and Western blotting, which are more sensitive and faster.^5^ For instance, when antibody titers measured in cerebrospinal fluid (CSF) are higher than serum antibody titers, it helps in the diagnosis of sporotrichosis meningitis. However, there is no association between the severity and distribution of lesions with the amount or type of the recognized antigen. Moreover, serological tests are still scarcely available in routine clinical practice.^54^, ^55^, ^56^

### Molecular tests

The limitations of traditional diagnostic methods have led to the development in recent years of some diagnostic methods at a molecular level, aiming to identify each species of *Sporothrix spp* through the “barcode” sequencing of its specific genes, improving the specificity, precision and sensitivity of the diagnosis.^52^

The protein-coding genes considered as markers for the identification, taxonomy, typing and epidemiology of *Sporothrix* species are beta-tubulin (BT2), elongation factor 1α (EF-1α), ITS1 and ITS2 (internal transcribed spacer), and calmodulin (CAL). CAL has been reported as the best marker and the most phylogenetically informative locus, being considered the gold standard.^57^

There are several studies in the literature describing the techniques that were used, and among them the most often used: restriction fragment length polymorphism (RFLP) of different target genes, nested PCR, random amplified polymorphic DNA (RAPD), DNA sequencing of the internal transcript spacer (ITS) of the rRNA, polymerase chain reaction (PCR) targeting the topoisomerase II enzyme gene, amplified fragment length polymorphism (AFLP), and M13 PCR fingerprinting. Most studies performed with these techniques have some limitations: they were performed on culture isolates and few on clinical samples, and the techniques are complex and time-consuming.^57^, ^58^, ^59^, ^60^, ^61^, ^62^

In 2012, de Oliveira et al. described the application of PCR fingerprinting using the universal primer T3B to differentiate between species of the *Sporothrix schenckii* complex (*S. brasiliensis*, *S. globosa, S. mexicana* and *S. schenckii*) and confirmed through the analysis of the partial sequence of the calmodulin (CAL) gene.^61^ Later, in 2015, they used the same methodology and added the differentiation of the *S. pallida* and *S. luriei* strains. This technique has the advantages of being simple, reliable, fast, and low-cost, making it an ideal identification system for use in clinical mycology laboratories that lack the resources for DNA sequencing.^62^

In 2019, Zhang et al. developed a real-time multiplex PCR, also based on the calmodulin gene sequencing, with 100% positive detection rates for *S. brasiliensis*, *S. schenckii*, and *S. globosa*, being a promising test for the marketing of a kit that is effective, fast, accurate, and highly sensitive.^60^

The relevance of the identification of *Sporothrix* species is due to their different characteristics, mainly the greater virulence of *S. brasiliensis*, followed by *S. schenckii* and *S. globosa*, which develop the most severe clinical forms. Additionally, it allows the tracking of disease transmission routes during epizootics and zoonoses in Brazil, associating specific genotypes to antifungal susceptibility profiles, as well as linking clinical findings of feline sporotrichosis with human sporotrichosis.^63^

### Other exams

There are intradermal tests that use sporotriquin or peptide rhamnomannan (PRM) antigen, which helps detect delayed hypersensitivity but are not sensitive or specific.^51^ Imaging studies such as radiography or computed tomography are requested when specific systemic involvement is suspected (for instance, in pulmonary sporotrichosis or osteoarticular sporotrichosis). They will provide support to the management of sporotrichosis but do not improve the specificity of the diagnosis of the disease.

## Treatment – available therapeutic arsenal, surgical intervention and management of hypersensitivity reactions

Although there have been reports of spontaneous regression of the classic form of sporotrichosis, it has not occurred with zoonotic sporotrichosis to date. In most cases, pharmacological treatment is essential.^64^ The prognosis of sporotrichosis in its classic form is excellent when the diagnosis is established and treatment is started early. However, there have been cases of the zoonotic form that linger for months or even years, despite adequate treatment.^26^, ^39^, ^48^

The pharmacological therapeutic arsenal for the treatment of sporotrichosis is limited. The therapeutic choice is primarily based on low cost, drugs that are easily administered, safety profile, and site and extent of the infection (whether localized or disseminated).^64^ Treatment duration varies. For mild cases, it takes an average of two to three months and can last up to one year in disseminated or systemic forms. The treatment must be maintained until the clinical cure is attained, that is, until the absence of signs that indicate disease activity, such as the presence of pus, exudation, and crusts on the skin lesions.

### Itraconazole

Itraconazole is used as the first-line drug to treat localized or disseminated cutaneous cases of sporotrichosis, as it promotes high clinical efficacy, safety, and few side effects, in addition to convenient dosing.^36^, ^64^ The dose varies according to disease severity and therapeutic response, from 100 to 400 mg/day, taken orally once or twice daily, until a clinical cure is attained, ranging from three to six months, and it can take up to one year in osteoarticular cases and disseminated forms. Treatment with 100 mg/day is effective in most mild cases, including the atypical forms.^3^, ^42^ Higher doses are usually recommended for poor responders or recurrence; however, they are accompanied by a higher frequency of adverse effects, such as headaches, gastrointestinal disorders, hypercholesterolemia, hypertriglyceridemia, liver function alterations, and peripheral edema, with the first two being the most frequently reported side effects. In children, the recommended dose is 5 mg/kg/day.^35^, ^50^ In cases with bone involvement, the recommended dose is 400 mg/day.^48^

It can be administered continuously or intermittently. Song et al., through a prospective randomized study, compared daily oral therapy (200 mg) with pulse therapy of itraconazole (400 mg/day, seven days/month), in 25 patients diagnosed with cutaneous sporotrichosis in each group. Although the patients who received the continuous oral therapy showed a higher cure rate after 48 weeks (95.8%×81.8%), the results were not statistically significant. This modality is not yet part of the consensuses, and further studies are required on pulse therapy in the treatment of sporotrichosis.^65^

Complete blood count, biochemistry, and liver function tests should be performed before treatment and after three to four weeks. If serum levels are within normal limits, the tests should only be repeated at the end of treatment.^35^

Its biggest disadvantage is drug interaction due to its metabolism dependent on the cytochrome P450 system, also common to other drugs such as phenytoin, simvastatin, and oral hypoglycemic agents. Therefore, its concomitant administration with drugs also metabolized by this enzyme is contraindicated due to potentially severe toxicity. Itraconazole is hepatotoxic and teratogenic and cannot be used by patients with liver disease or pregnant women (risk category C).

### Potassium iodide

Potassium iodide, which has historically been the treatment of choice for sporotrichosis, is an effective and low-cost option, which is of great value in endemic areas of developing countries and in cases that do not respond to itraconazole. However, it is not effective in the extracutaneous forms of sporotrichosis.^64^

It is usually administered orally in a saturated solution of potassium iodide. In the saturated solution, each drop contains 0.07 g, and in the concentrated solution, 0.05 g of potassium iodide. The recommended dose in adults is five drops three times a day, gradually increasing three drops per daily dose until the highest well-tolerated dose is reached, which is approximately 30 to 40 drops three times a day. Its administration with fruit juice or milk can mitigate the unwanted gastrointestinal effects. For children, it is recommended to start with three drops three times a day, increasing one drop/kg per dose to a maximum of 25 drops three times a day.^50^

A recent study has shown that potassium iodide, in the form of a saturated solution, administered at a reduced dose and frequency, can be used as an effective and safe alternative for the treatment of cutaneous sporotrichosis. Doses of 1 to 2 g/day for children and 2 to 4 g/day for adults, given three times daily, are effective in curing most patients.^66^

The mechanism of action of KI in sporotrichosis remains unknown, despite its known action in inhibiting the formation of granulomas, phagocytosis of *Sporothrix* cells, as well as in immune response. This immunomodulatory effect also makes it useful in immunoreactive presentations of sporotrichosis. Its use is limited by its adverse effects, which include gastrointestinal disturbances, metallic taste, coryza, rash, salivary gland enlargement, and thyroid disturbances. Its prolonged use, especially in patients who already have a defect in thyroid function regulation, can lead to the interruption of the endogenous production of thyroid hormones, a phenomenon known as the Wolff-Chaikoff effect. For this reason, it is contraindicated in patients with thyroid dysfunction, as well as in pregnant women (category D) and lactating women. In case of toxicity, the dose should be reduced until there are no more adverse effects. ^66^

### Terbinafine

Terbinafine at a dose of 250 mg/day can be used in the cutaneous forms of sporotrichosis, with a cure rate greater than or equal to that of itraconazole 100 mg/day.^67^ It can be prescribed in cases that do not respond to itraconazole or when the latter is not well tolerated or contraindicated, as well as in patients who use statins, since it has a low binding to cytochrome P450 and does not interfere with the bioavailability of other drugs, unlike itraconazole. For this reason, it is also useful in the treatment of elderly patients with comorbidities.^67^ This drug is considered category B for pregnant women.

### Fluconazole

Fluconazole can be used as an alternative for resistant forms of sporotrichosis, at a dose of 400 mg/day for three to six months. As the experience with fluconazole in the treatment of sporotrichosis is limited and it shows moderate effectiveness, it is considered a second-line treatment option for those patients who are intolerant to itraconazole. Fluconazole is contraindicated during pregnancy due to its teratogenic potential.^64^

### Amphotericin B

Amphotericin B is the drug of choice in the treatment of severely compromised patients, such as cases of disseminated, pulmonary, meningeal sporotrichosis, or immunocompromised patients. It may also be indicated for extensive osteoarticular sporotrichosis or cases unresponsive to itraconazole.^64^

The recommended dose of amphotericin B is 0.5 to 1 mg/kg daily until a favorable clinical response is achieved, followed by itraconazole 400 mg daily as maintenance therapy. After clinical stabilization, it is recommended to change the medication to itraconazole 200 mg daily for a minimum of 12 months.^64^

The literature lacks substantial data for choosing the lipid formulation of amphotericin over deoxycholate, with the exception that the lipid formulation could be preferred in cases of meningitis. There are animal data showing that higher concentrations are achieved in brain tissue with the lipid formulation of amphotericin B when compared to the deoxycholate form. However, the relevance of this discovery for the treatment of meningeal sporotrichosis remains unknown. Moreover, it is possible to use higher doses (3–5 mg/kg/day) with the lipid formulations, with less toxicity.^64^

### Thermotherapy

The daily application of local heat on the skin lesion, using warm compresses or devices that emit infrared waves, is recommended for the treatment of small lesions and a more acceptable form of treatment in pregnant women and patients intolerant to oral antifungal agents. Thermotherapy can be combined with pharmacological treatment, and it is recommended to apply local heat for 15 minutes to one hour several times a day, until complete resolution of the condition or until the introduction of drug treatment.^3^, ^64^

### Surgical intervention

Lobectomy can be considered in pulmonary sporotrichosis, segmental cases, or in those unresponsive to treatment.^38^ The combination of surgical resection and amphotericin B in these cases is considered the best therapeutic approach and is superior to the use of these measures alone.^64^

Cryosurgery with liquid nitrogen can be used as a therapeutic complement in refractory cases of the fixed form of sporotrichosis or the cutaneous lymphatic form. This method should be recommended, particularly when the lesions have crusts and are infiltrated, as well as in isolated cases of localized lesions in immunocompetent patients.^68^ Electrosurgery^69^ and surgical removal constitute other valid therapeutic options in selected cases with lesions that have not responded to conventional drug therapy. All therapeutic methods described can be used as monotherapy or as adjuvant therapy. Generally, the therapeutic response is more effective when two therapeutic modalities are combined, especially when it involves an associated surgical method.

### Management of hypersensitivity reactions

Hypersensitivity reactions reported in the literature were mostly treated with oral corticosteroids at different doses, depending on the extension of the picture and symptom intensity. The most frequent of these reactions, Sweet’s syndrome, has responded very well to corticosteroid therapy.^43^ Some authors have reported improvement in the clinical picture only with the correct use of antifungal treatment. Gutierrez-Galhardo et al. demonstrated the favorable evolution of erythema nodosum associated with sporotrichosis using the standard treatment for mycosis (itraconazole 100 mg/day).^42^ Likewise, the use of non-hormonal anti-inflammatory drugs has also been reported for the treatment of milder reactions.^45^

## Perspectives

Recent studies involving two important components of the fungus cell wall, glycoproteins 60 kDa and 70 kDa, show that they act as a virulence factor and play an important role in the adhesion and immunomodulation of the fungus. Monoclonal antibody P6E7 IgG1 against gp70 is potentially useful for disease therapy because it has shown a significant reduction in the host fungal load, thus preventing the adhesion of *Sporothrix* to extracellular matrix components. This robust protection makes it a strong candidate for a therapeutic vaccine against sporotrichosis.^35^

## Financial support

None declared.

## Authors’ contributions

Regina Casz Schechtman: Design and planning; drafting and editing; effective participation in research orientation; critical review of the literature; critical review of the manuscript; intellectual participation in the propaedeutic and/or therapeutic conduct of the studied cases; approval of the final version of the manuscript.

Eduardo Mastrangelo Marinho Falcão: Drafting and editing of the manuscript; collection, analysis, and interpretation of data; effective participation in research orientation; critical review of the literature; critical review of the manuscript; intellectual participation in the propaedeutic and/or therapeutic conduct of the studied cases; approval of the final version of the manuscript.

Marciela Carard: Drafting and editing of the manuscript; collection, analysis, and interpretation of data; critical review of the literature; approval of the final version of the manuscript

Maria Salome Cajas Garcia: Drafting and editing of the manuscript; critical review of the literature; approval of the final version of the manuscript

Diana Stohmann Mercado: Drafting and editing of the manuscript; critical review of the literature; approval of the final version of the manuscript.

Roderick James Hay: Design and Planning of the study; critical review of the literature; Drafting and editing of the manuscript; Critical review of the manuscript; Approval of the final version of the manuscript.

## Conflicts of interest

None declared.
